# Feasibility of second-generation bioresorbable vascular scaffold implantation in complex anatomical and clinical scenarios

**DOI:** 10.1007/s00392-014-0757-4

**Published:** 2014-08-31

**Authors:** Milosz Jaguszewski, Jelena-Rima Ghadri, Manuel Zipponi, Dana Roxana Bataiosu, Johanna Diekmann, Verena Geyer, Catharina Anna Neumann, Mia Aurelia Huber, Christian Hagl, Paul Erne, Thomas F. Lüscher, Christian Templin

**Affiliations:** 1 University Heart Center, University Hospital Zurich, Raemistr. 100, 8091 Zurich, Switzerland; 2 Faculty of Law, University of Zurich, Zurich, Switzerland; 3 Department of Heart and Thoracic Surgery, Ludwig-Maximilians University Munich, Munich, Germany; 4 Department of Cardiology, Kantonal Hospital of Lucerne, Lucerne, Switzerland

**Keywords:** Bioresorbable vascular scaffold, Optical coherence tomography

## Abstract

**Background:**

Bioresorbable vascular scaffolds (BVS) have become an emerging tool to treat coronary artery disease. However, the current use of BVS is still widely restricted to stable patients and non-complex lesions. In real-world practice patients are far more complex than those with simple type A lesions and the extended use of BVS to complex lesions and high-risk patients needs to be evaluated. Therefore, we sought to investigate the feasibility and performance of BVS in a broad spectrum of patients.

**Methods:**

106 patients underwent in total 193 BVS implantations. We assessed the device-related (cardiac death, target vessel myocardial infarction, ischemia-driven target lesion revascularization) and patient-related (all-cause death, any reinfarction and any revascularization) composite outcomes.

**Results:**

90 % of patients (*n* = 95) had at least one of the following characteristics: >65 years (35 %), ACS (42 %), tortuous vessels (13 %), calcified (17 %) or thrombotic lesions (12 %), lesions defined as AHA type B2/C (42 %), bifurcations (16 %), chronic total occlusions (9 %) or restenosis (14 %). There was no evidence of significant edge dissection, huge thrombus load or incidence of scaffold dislodgement or scaffold disruption in optical coherence tomography pullbacks. Out of 10,157 struts evaluated within 1,117 cross-sections, 302 were classified as malapposed (2.9 %). During a mean follow-up of 147 ± 119 days the rate of device-related events was 2.0 %, whereas patient-related composite events occurred in 6.1 %.

**Conclusions:**

Our results strongly suggest that BVS implantation is feasible in a wide spectrum of patients and complex anatomy of coronary lesions. Long-term outcome of BVS should be further investigated in unrestricted settings in randomized controlled trials.

**Electronic supplementary material:**

The online version of this article (doi:10.1007/s00392-014-0757-4) contains supplementary material, which is available to authorized users.

## Introduction

The first plain-old balloon angioplasty (POBA) performed by Andreas Gruentzig in 1977 in Zurich, Switzerland, revolutionized the treatment strategy of coronary artery disease (CAD) [1]. The common acute and chronic recoil was reduced by the subsequent introduction of bare-metal stents (BMS), as reported in the BENESTENT trial [2]. However, as the restenosis rate remained high with BMS, drug-eluting stents (DES) were developed. Although the first-generation DES essentially reduced the need for urgent revascularization as compared to BMS, their propensity for late stent thrombosis (ST) was a concern [3]. Furthermore, the need for target lesion revascularization after DES implantation remains a still not fully resolved issue. The design of new second-generation DES with biocompatible polymer coatings allowed for improved deliverability, endothelial healing and therefore better device-rated outcomes [4]. Nowadays, the new technology of bioresorbable vascular scaffolds (BVS) remains a ray of hope in the field of interventional cardiology. Nonetheless, data are still lacking regarding their use in a real-world setting. Indeed, so far these novel scaffolds have been exclusively used in stable patients with non-complex lesions [5–8]. Currently, only little data exist regarding real-world use of these devices, i.e., in patients with acute coronary syndromes (ACS), chronic total occlusions (CTO), bifurcations, and restenosis [9–13]. We therefore aimed to investigate the clinical outcome after BVS implantation in two highly experienced centers in a wide patient spectrum.

## Methods

### Population, data collection, and procedures

**Fig. 1:**
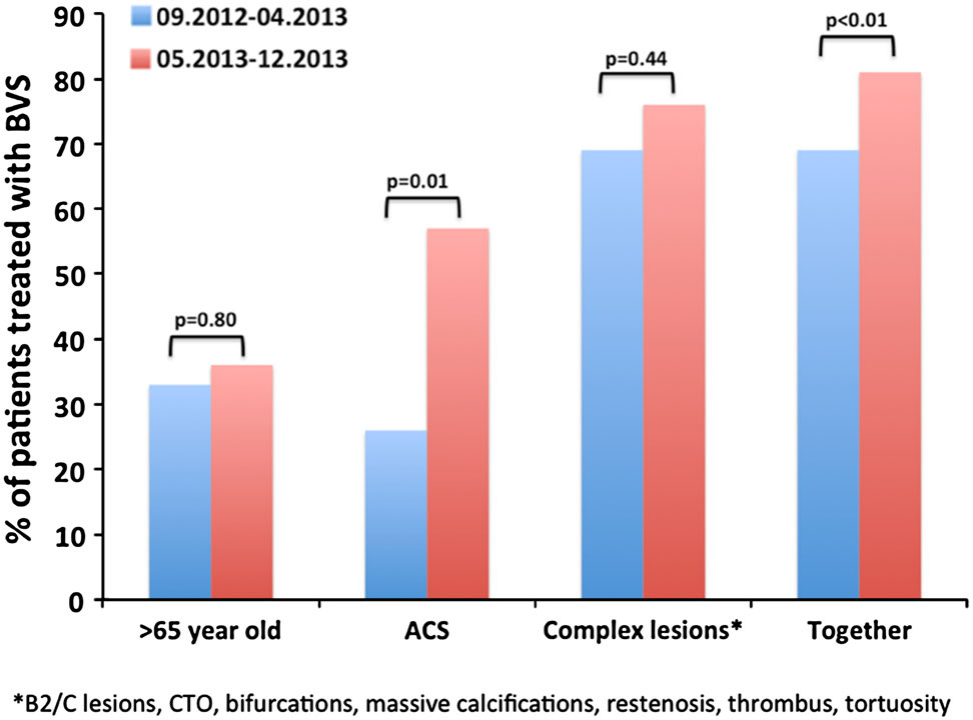
Temporal trends in patients’ age and lesion characteristic for the usage of BVS

The study flowchart is presented in Fig. 1. Patients from two Swiss tertiary cardiology centers (University Heart Center, Cardiology, Zurich and Cardiology, Regional Hospital Lucerne) with stable CAD or ACS who qualified for percutaneous coronary intervention (PCI) were enrolled from September 2012 to December 2013. Eligible patients had at least one coronary artery lesion and no restrictions as to the number, severity and location of stenosis. All patients were implanted with at least one second-generation BVS (Abbott Vascular, Santa Clara, California) designed with semicrystalline poly-l-lactide (PLLA) and coated with an amorphous poly-d, l-lactide (PDLA) polymer eluting everolimus. The reason for the selection of BVS was left to the discretion of the operator. Medical records including demographics, clinical, angiographic and procedural features were collected with the use of special electronic databases. The use of anonymized data, originating from retrospective analysis of personal data from own patients exclusively, requires no approval by the local ethics committee.

The intervention was obtained according to the current PCI guidelines. All lesions were prepared with the use of balloon dilatation before each BVS implantation and BVS deployment was performed in line with the manufacturer’s recommendations at a rate of 2 atmospheres per 5 s up to rated burst pressure. If more than one BVS was implanted, the overlap technique or adjacent-positioning technique was used.

Coronary angiography was performed in the left coronary system with two radiation exposures in four projections and in the right coronary artery with two exposures in two projections. Lesions were classified according to the AHA/ACC criteria as follows—type A: minimally complex, concentric, readily accessible of length <10 mm in non-angulated segment (<45°), with little or no calcification, less than totally occlusive, not ostial in location, with no major side branch (SB) involvement, and no apparent thrombus; type B1: moderately complex, with a length of 10−20 mm, eccentric and with irregular contour, moderate or heavy calcification in a proximal segment of moderate tortuosity, moderately angulated (>45 and <90°), total occlusions <3 months old, ostial in location, and bifurcation lesions requiring double guidewires with some thrombus present; type B2: lesions demonstrating two or more type B1 characteristics; type C: severely complex, with length of >20 mm, in a tortuous, proximal, extremely angulated segment, total occlusions >3 months old and/or bridging collaterals, and degenerated vein grafts.

Patients were treated with dual antiplatelet therapy (DAPT) comprising a loading dose of aspirin 500 mg i.v., followed by the maintained daily dose of 100 mg, and clopidogrel 600 mg p.o. followed by the maintained daily dose of 75 mg for 12 months or prasugrel 60 mg p.o. continued with 10 mg daily dose or ticagrelor 180 mg loading dose and continued with 2 × 90 mg daily dose. Heparin was administered intravenously to maintain the activated clotting time of at least 250 s. The use of thrombectomy and GP IIb/IIIa administration was at the discretion of the respective cardiologist. Manual thrombectomy was performed using an Export catheter (Medtronic Inc., Minneapolis, USA).

### Definitions

ST-segment elevation myocardial infarction (STEMI) was defined as a new ST-segment elevation of at least 1 mm in two or more contiguous leads or new left bundle branch block (LBBB) concomitant with elevated troponin level [14]. Non-ST-segment elevation myocardial infarction (NSTEMI) was defined as the elevation of biomarkers of myocardial necrosis (e.g., troponin) with no evidence of ST-segment elevation in the ECG in the presence of angina chest pain [15]. CTO was defined as a complete obstruction of coronary artery with thrombolysis in myocardial infarction (TIMI) flow grade 0 and an estimated time of occlusion of at least 3 months [16]. Target lesion revascularization (TLR) was defined as repeat intervention in a de novo lesion within the stented segment with a luminal diameter of ≥50 % or within a 5 mm border zone proximal or distal to the stent. An “off-label indication” was defined as a BVS implantation in patients >65 years, those referred for primary PCI due to an ACS, in tortuous vessels, calcified or thrombotic lesions, lesions defined as AHA type B2/C, bifurcation lesions, CTO, and in restenosis.

### Optical coherence tomography (OCT)

The C7-XR OFDI system (LightLab Imaging, Inc, St Jude Medical, St. Paul, MN, USA) was optionally used to acquire images during intracoronary injection of contrast media with an optical fiber Dragonfly or Dragonfly Duo catheter. The Dragonfly catheter was positioned over a conventional 0.014′ angioplasty guidewire and automated pullback (20 mm/s), with an image acquisition at 100 frames/s, and contrast injection (6 mL/s) was used to acquire all images.

All pullbacks were analyzed post hoc in our core laboratory of the Andreas Gruentzig Catheterization Laboratories at the University Heart Center, University Hospital of Zurich, Switzerland using proprietary software (ILUMIEN OPTIS system, Inc, St Jude Medical, St. Paul, MN, USA). The analysis was performed in 1 mm longitudinal intervals within treated segments and 5 mm proximal and distal. Image quality was assessed using a four-point scale (excellent, good, moderate, non-diagnostic) and only excellent pullbacks, i.e., with >95 % of cross sections with full lumen contour visibility, were included in the final analysis. All BVS were investigated with regard to strut apposition, strut fracture, edge dissections, and thrombus formations. Every single strut was analyzed in each OCT frame. Struts were considered malapposed when the abluminal surface was separated from the vessel wall.

Three-dimensional reconstructions of OCT were performed for an optimal visualization of bifurcations. Ostia of all side branches of >1.5 mm diameter were assessed visually and classified as follows: non-jailed SB, when no compartments were present in the SB orifice; jailed SB, when BVS struts separated the orifice of SB into compartments with different patterns (V, T, H, and double T) as described previously [17].

Edge dissection of >30 % of lumen circumference was considered as significant. Procedural strut fracture was defined if two struts overhung each other in the same angular sector of the lumen and/or if struts were located in the lumen without any connection with other struts [18].

The use of OCT was left at the discretion of the operator and was especially used in case of a large thrombus burden, in STEMI patients, bifurcations, and to track the BVS implantation after CTO recanalization. We intended to avoid OCT evaluation in patients with hemodynamic deterioration and chronic renal failure. OCT evaluation was performed by the investigator blinded to the clinical characteristics and outcome.

### SYNTAX score

SYNTAX score assessment was performed by a cardiologist blinded to the clinical characteristics and outcomes of the patients using a scoring system for all significant lesions (≥50 %) in the vessels ≥1.5 mm in diameter using the SYNTAX score algorithm. The SYNTAX score was calculated using angiography just after the first dilatation of the culprit vessel, thereby allowing inclusion of all significant lesions [19].

### Outcomes

Follow-up analysis included device-related composite outcome measures of cardiac death, target vessel myocardial infarction, ischemia-driven TLR, and patient-related outcome measure of all-cause death. Any reinfarction and any revascularization was collected routinely by using standardized clinical questionnaire for hospital quality assessment. Furthermore, we evaluated the rate of ischemia-driven TLR and definite/possible/probable scaffold thrombosis.

### Statistics

Data are presented as percentages mean ± SD. Categorical variables were compared with Chi square test. A *p* value < 0.05 was considered to be statistical significant.

## Results

### Clinical characteristics

Patients’ baseline characteristics are presented in Table 1. The average age was 61.0 ± 10.7 years with only 35 % patients (*n* = 37) being older than 65 years. PCI was successfully performed in all patients (*n* = 106). Full revascularization was obtained in 87 % of patients (*n* = 92). Mean left ventricular ejection fraction was 59.3 ± 13.3 %. Four percent of patients (*n* = 4) required GP IIb/IIIa infusion and in 13 % (*n* = 14) manual thrombectomy was performed. The study flowchart has been presented on Supplementary Fig. 1.

**Table 1:** Baseline characteristics

	Baseline characteristics (*n* = 106)
Age	61.0 ± 10.7
Gender (male)	81 (76.4)
BMI (kg/m^2^)	27.5 ± 3.5
Stable angina	62 (58.5)
ACS	44 (41.5)
STEMI	18 (17.0)
Cardiovascular history	
Prior Ml	16 (15.1)
Stroke	4 (3.8)
Cardiovascular risk factors	
Hypertension	47 (44.3)
Hyperlipidemia	56 (52.8)
DM	21 (19.8)
Smoking, current	34 (32.1)
Obesity	22 (20.8)
FH	43 (40.6)
Hemodynamics	
LVEF	59.3 ± 13.3
LVEDP	17.9 ± 7.3
HR	71.5 ± 15.5
SBP	130.8 ± 23.3
DBP	70.7 ± 10.6
Adjunctive therapy	
Thrombectomy	14 (13.2)
GP	4 (3.8)

Depicted are counts, *n*, incidence (%) or mean ± SD

*ACS* acute coronary syndrome, *BMI* body mass index, *DBP* diastolic blood pressure, *DM* diabetes mellitus, *FH* known family history, *GP* glycoprotein llb/llla inhibitor, *HR* heart rate, *LVEF* left ventricle ejection fraction, *LVEDP* left ventricle end-diastolic pressure, *MI* myocardial infarction, *SBP* systolic blood pressure, *STEMI* ST-segment elevation myocardial infarction

### BVS implantation and OCT

**Fig. 2:**
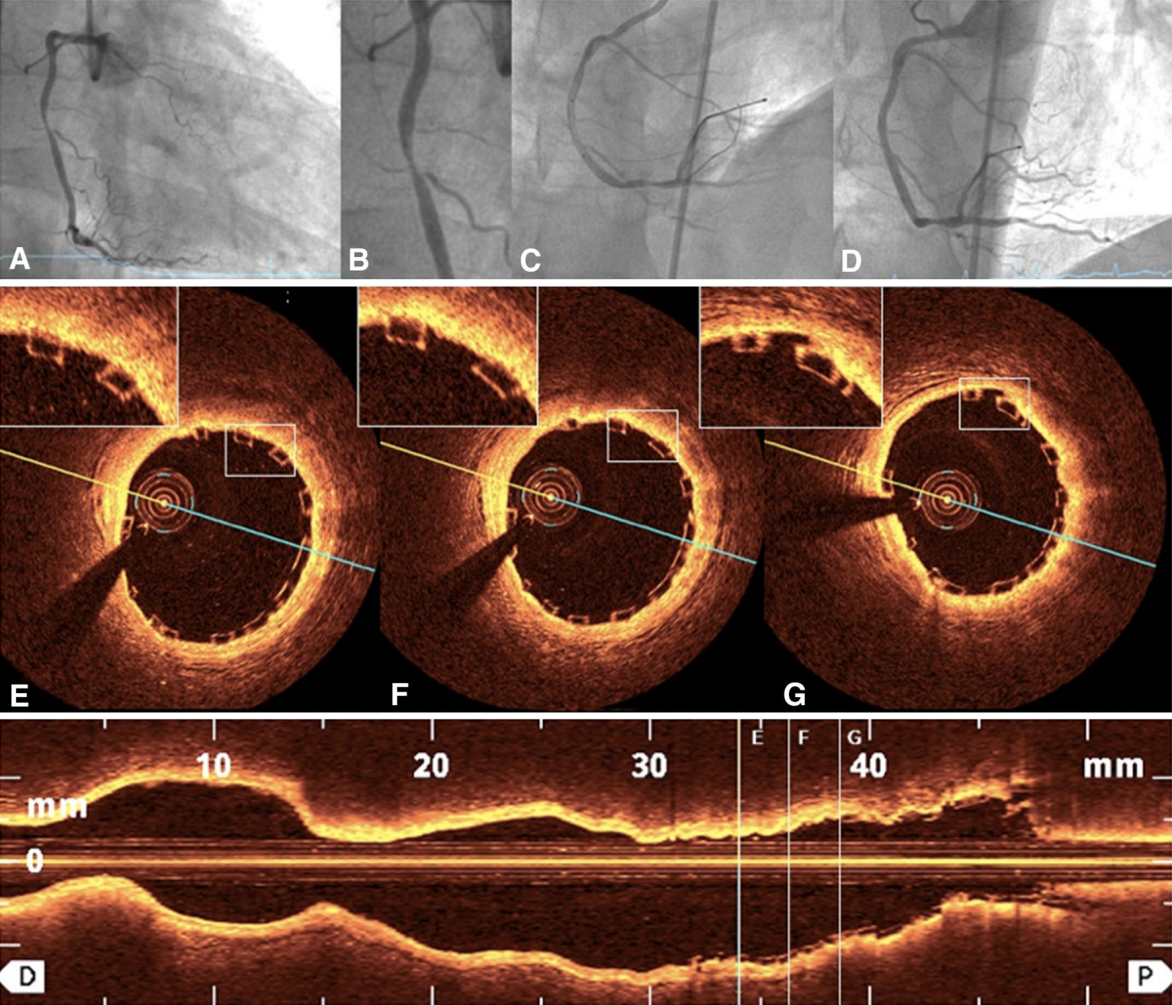
BVS in type A lesion. Type A lesion in the middle portion of the right coronary artery (**a** and **b**) predilated with a 3.0 × 15 mm balloon and subsequently covered by 3.5 × 20 mm BVS (**c**) with excellent angiographic result (**d**). The OCT pullback documented well-apposed struts in all cross sections (**e**−**g**)

In total, 193 BVS were implanted with an average amount of 1.82 ± 1.19 per patient and average length of 42.6 ± 30.7 mm per patient. The minimal length of implanted BVS was 18 mm and the maximal length 186 mm (7 BVS in one patient). The scaffold diameters used were 2.5, 3.0 and 3.5 mm. The mean SYNTAX score was 14.4 ± 10.6 (range 1−51). The implantation of BVS was extended to more acute situations with increasing experience of the operators (Fig. 1). Out of 139 lesions covered by BVS, 25 % (*n* = 34) were defined as type A (Fig. 2) and 34 % (*n* = 47) as type B1. 90 % of patients (*n* = 95) had at least one of the following characteristics: >65 years (35 %), ACS (42 %), tortuous vessels (13 %), calcified (17 %) or thrombotic lesions (12 %), lesions defined by AHA type B2/C (42 %), bifurcations (16 %), CTO (9 %), or restenosis (14 %) (Table 2). Three patients with coronary dissection received BVS. The procedural success rate was 97 %. TIMI 3 flow grade was restored in all patients. Three BVS were not implanted due to the inability to pass the lesion. These patients were excluded from the final analysis. In two cases coronary artery rupture occurred and was successfully covered by stent graft implantation.

**Table 2:** Angiography characteristics

	Angioraphic characteristics (*n* = 106)
Angiography	
Single-vessel disease	44 (41.5)
Multivessel disease	62 (58.5)
SYNTAX score	14.4 ± 10.6
Full revascularization	92 (86.8)
TIMI flow 0 at baseline	22 (20.8)
TIMI flow 3 after PCI	106 (100)
Lesions treated by BVS	139
Type A	34 (24.5)
Type Bl	47 (33.8)
Type B2	34 (24.5)
Type C	24 (17.2)
BVS implanted per patient	1.82 ± 1.19
Unrestricted indications	
Chronic total occlusion	10 (9.4)
Restenosis	15 (14.2)
Bifurcations	17 (16.0)
Heavy calcifications	18 (17.0)
Thrombus	13 (12.3)
Tortuous vessel	14 (13.2)

Depicted are counts, *n* incidence (%) or mean ± SD

*TIMI t*hrombolysis in myocardial infarction

**Fig. 3:**
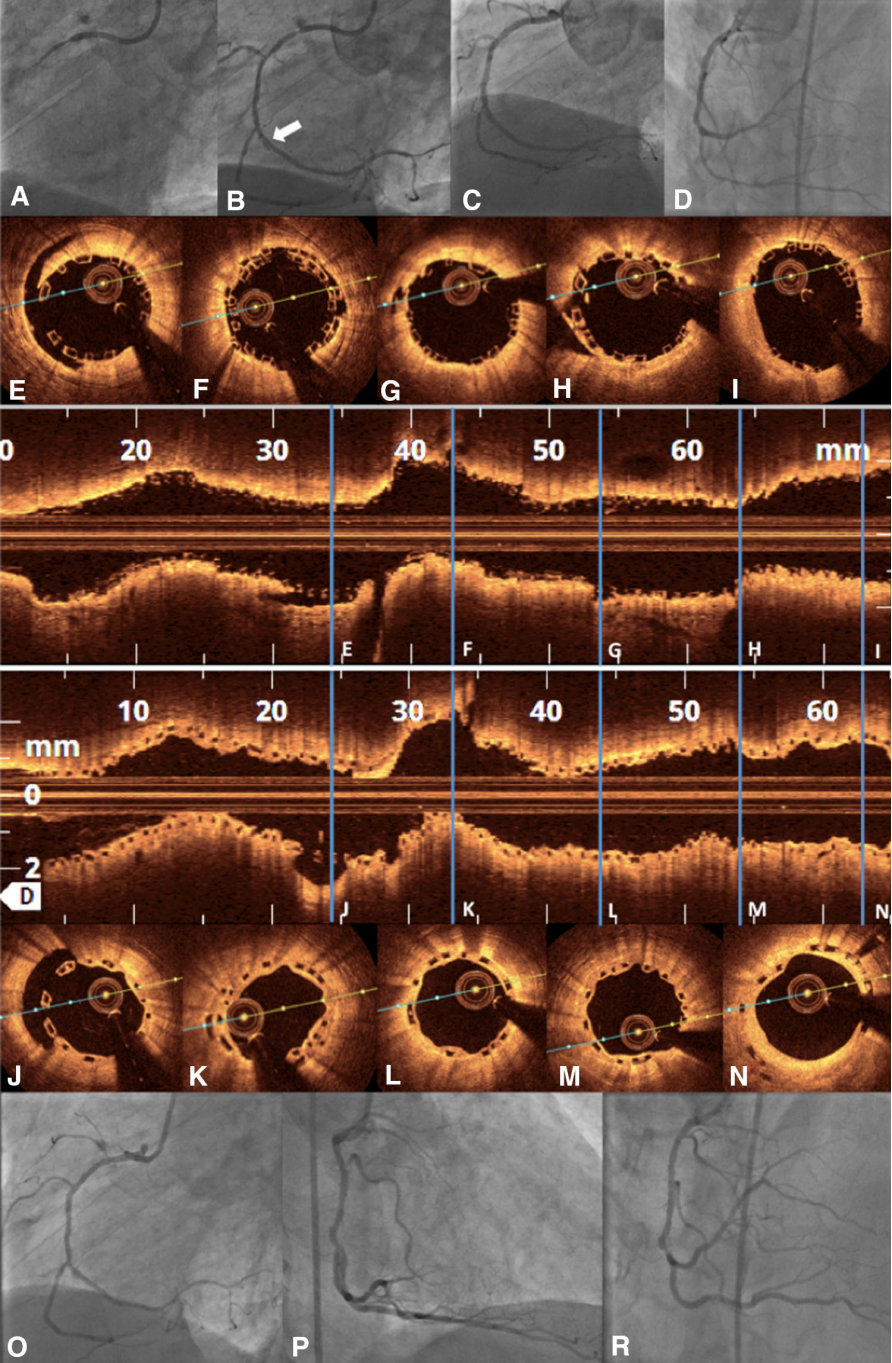
BVS in true chronic total occlusion. Elective recanalization of a chronic total occlusion of the right coronary artery (RCA) (**a**). After crossing the CTO with a pilot guidewire and an extensive vascular sealing with an Avion-Plus 1.25 × 20 mm balloon, Maverick 2.0 × 20 mm balloon and Maverick 2.5 × 30 mm balloon (**b**, dissection—*white arrow*), two BVS 2.5 × 28 mm were implanted in the distal and medial portion of the RCA. Subsequently, after catheter exchange (AL1-to-JR4-side-holes) and BMW guidewire placement, the predilatation of the proximal and ostial portion of the RCA was obtained with the use of a Maverick 3.0 × 15 mm balloon. Finally, the third BVS 3.0 × 18 mm was implanted to cover the ostial part of the RCA (**c** and **d**). The OFDI demonstrated an optimal postprocedural result (**d**−**l**) with well-apposed scaffold struts (**e**−**i**) and full coverage of iatrogenic dissections after the recanalization. The thickness of two overlapping struts was 300 μm (**f** and **i**, pullback—Supplemental video 1). The OFDI after 5-month follow-up demonstrated full coverage of BVS struts (**j**−**n**, pullback—Supplemental video 2) and an optimal result in angiography (**o**−**r**)

**Fig. 4:**
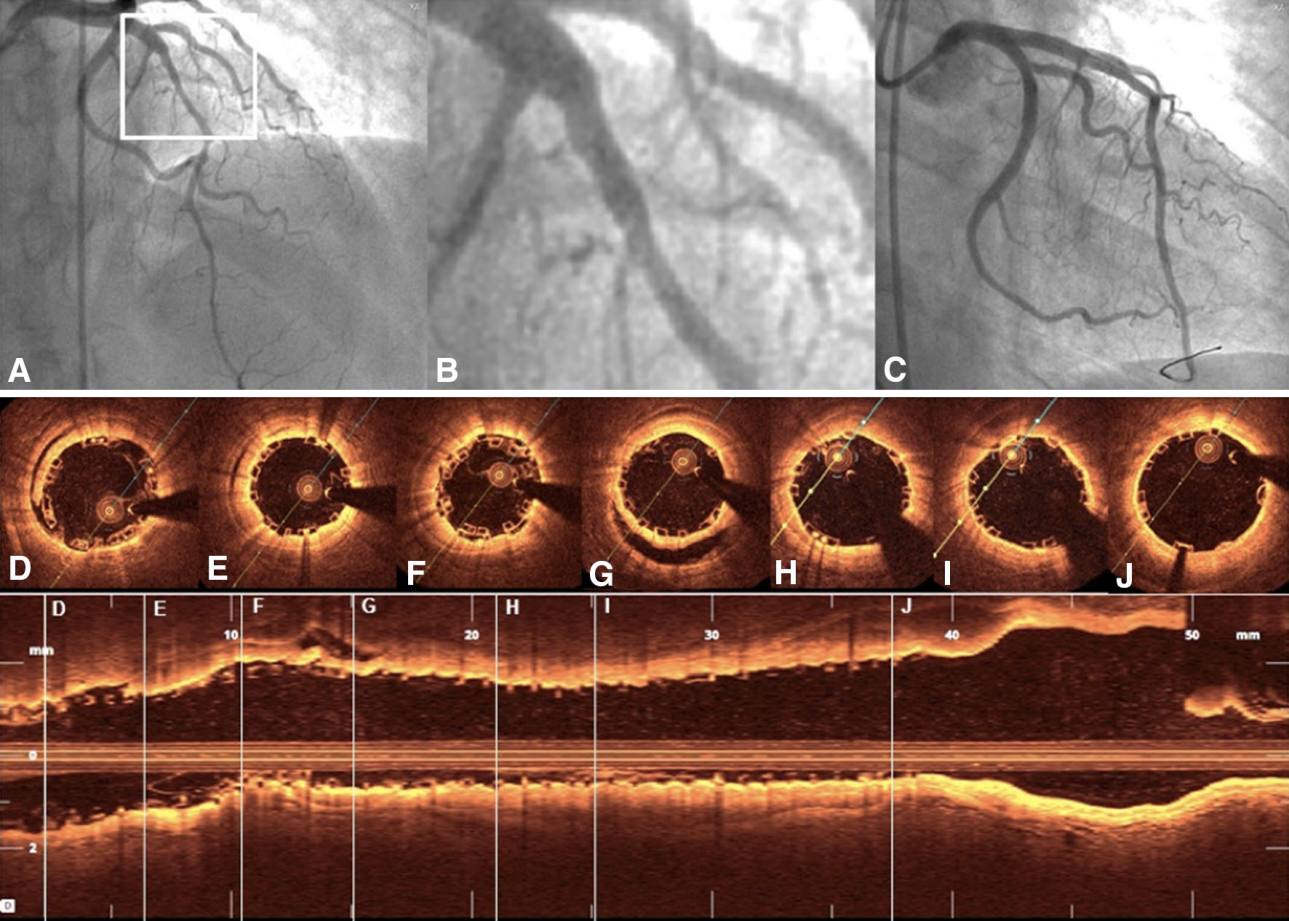
BVS in a long dissection in young adult. Non-ST segment elevation myocardial infarction with long dissection starting in the middle portion of the left anterior descending artery (LAD) (**a** and **b**). After thrombectomy and extensive dilatation with a 2.5 × 30 mm balloon, five BVS were implanted (three times 2.5 × 28 mm, 3.0 × 28 mm, and 3.5 × 28 mm) (**c**). The OCT documented a satisfactory result in the LAD (**d**−**j**); (pullback—Supplemental video 3)

**Fig. 5:**
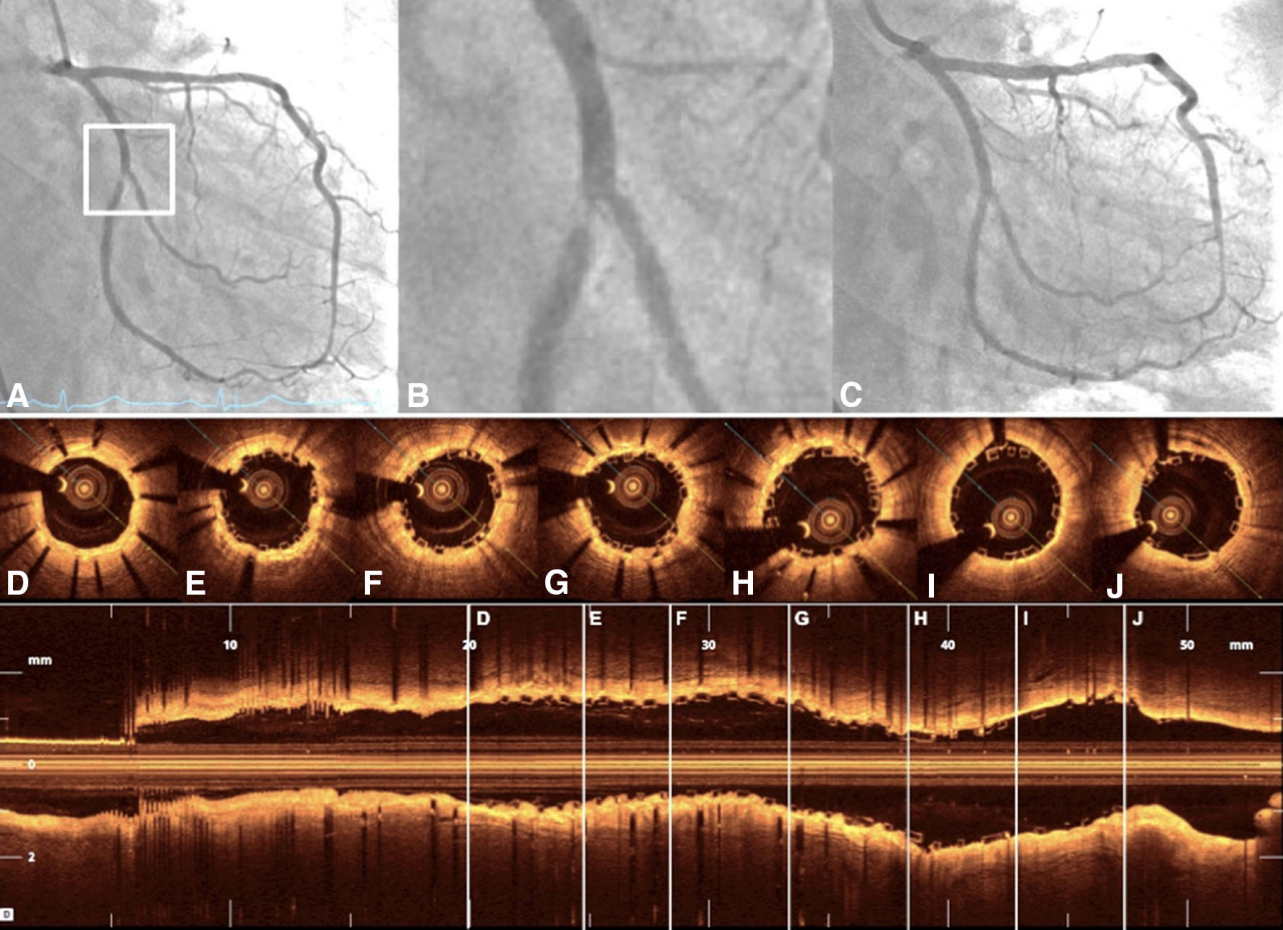
BVS in in-stent-stenosis. BVS implantation in a stent restenosis of a bifurcation lesion after biolimus-eluting stent implantation in the middle portion of the circumflex artery (LCx) (**a** and **b**). After extensive predilatation in the marginal branch and LCx with 2.5 × 15 mm and 2.5 × 30 mm drug-eluting balloons, a 2.5 × 28 mm BVS was implanted. Due to small edge dissection after BVS implantation, a second 3.0 × 18 mm BVS was used to cover the proximal portion of the LCx (**c**). OCT pullback recorded an excellent postprocedural result (**d**−**j**); (pullback—Supplemental video 4)

**Fig. 6:**
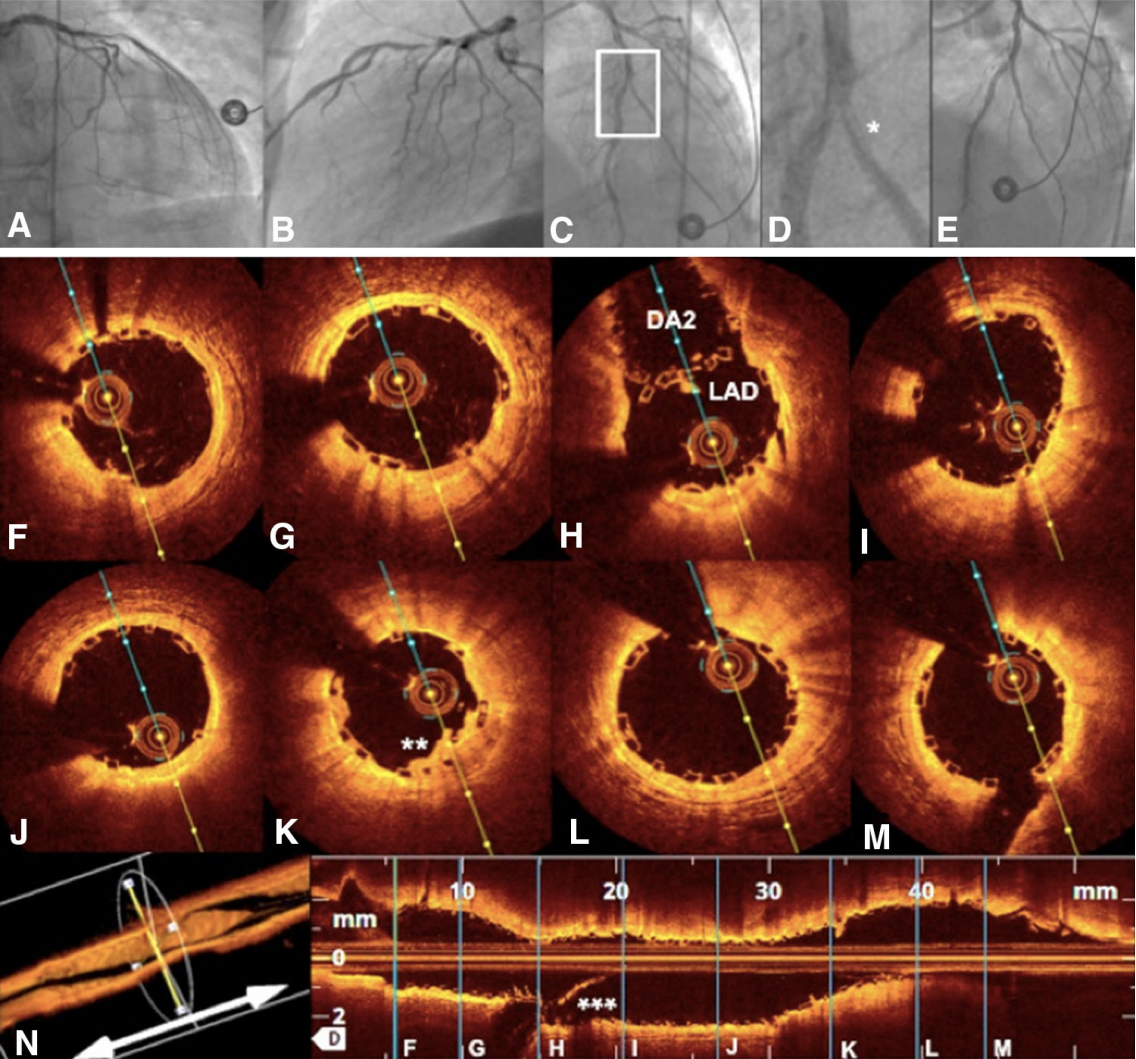
BVS-T stenting of a bifurcation lesion. Bifurcation lesion in acute coronary syndrome and slow flow in left anterior descending artery (LAD, **a** and **b**). After passing the lesion with a BMW guidewire, manual thrombectomy, and vascular sealing with a Maverick 2.5 × 15 mm balloon, a BVS 3.0 × 18 mm was implanted in the middle portion of the LAD and extended by a second BVS 3.0 × 12 mm. The second BMW guidewire was retrieved to the bifurcation of the second diagonal branch (DA2) and advanced again through the struts of the previously implanted BVS. After subsequent dilatation in the ostial portion of the DA2 with a Maverick 2.5 × 15 mm balloon, a BVS 2.5 × 12 mm was gently positioned through the BVS struts to seal the iatrogenic dissection (**c** and **d**). The final angiogram (**e**) and optical coherence tomography (OCT) (**f**−**n**) demonstrated an optimal postprocedural result with well-apposed struts in all cross sections (**e**−**m**); (pullback—Supplemental video 5). *Dissection in DA2; **protruding thrombi inapparent in angiography; ***BMW guidewire to DA2

**Fig. 7:**
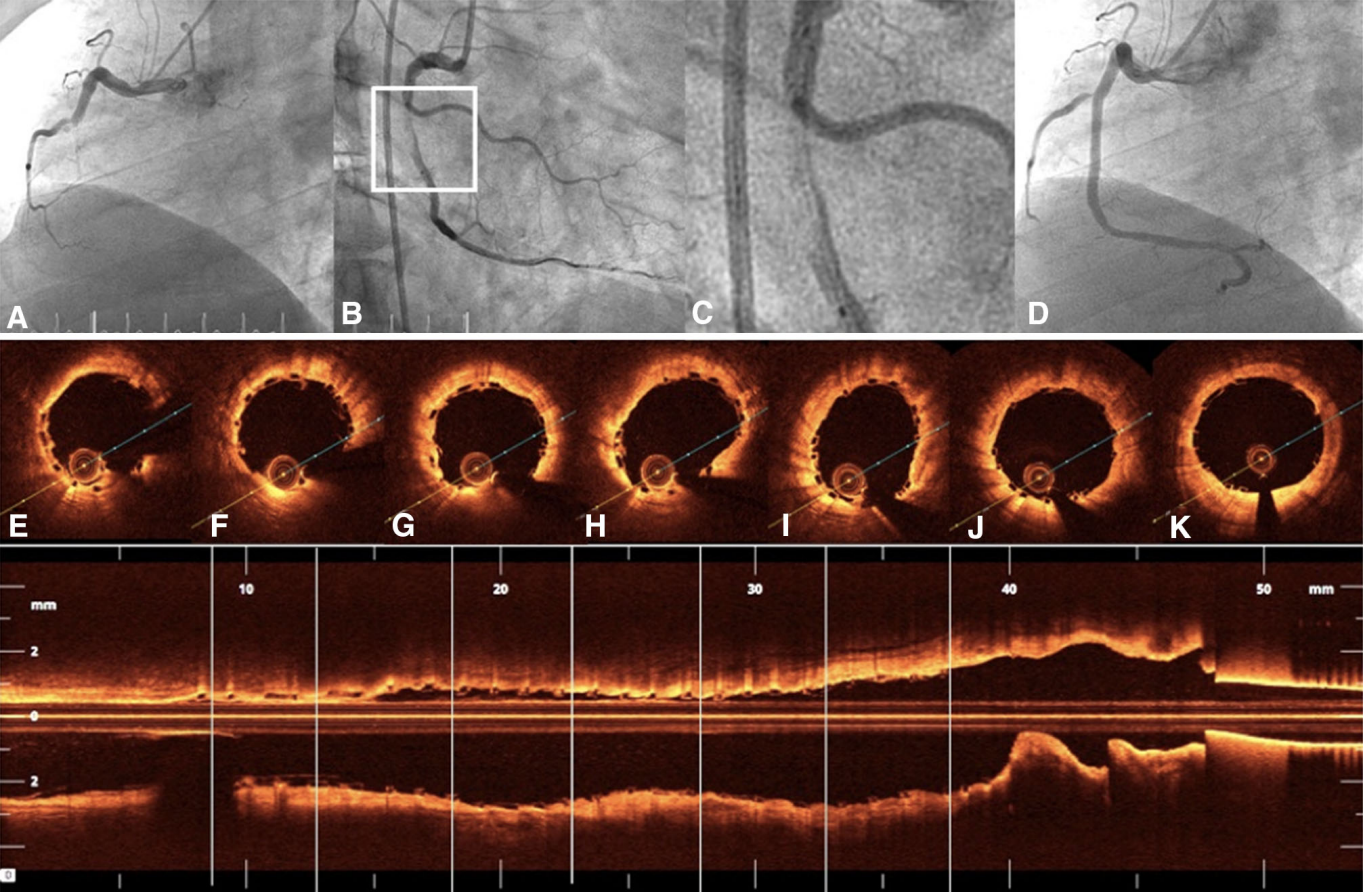
BVS in acute ST-segment elevation myocardial infarction. Right coronary artery occluded with a thrombus during the acute course of an inferior ST-segment elevation myocardial infarction (**a**). After manual thrombectomy with an Export catheter and extensive predilatation with a 3.0 × 15 mm balloon, a 3.5 × 28 mm BVS was implanted (**b** and **c**, BVS positioning). Final angiography cine (**d**) and OCT pullback documented an excellent result (**e**−**k**). *Residual adherent thrombi (pullback—Supplemental video 6)

OCT was performed at the end of PCI in 36 of the 106 patients (34 %) in which BVS implantation was considered most challenging and high risk, i.e., after CTO recanalization (Fig. 3), in long left anterior descending artery dissection (Fig. 4), in-stent restenosis (Fig. 5) and bifurcations (Fig. 6) and in patients with thrombotic occlusion during an STEMI event (Fig. 7). Six pullbacks were excluded due to the suboptimal imaging quality. All OCT pullbacks and cross-sections showed an excellent result after BVS implantation, with no evidence of significant postprocedural edge dissection, huge thrombus load, incidence of scaffold dislodgement, or scaffold disruption.

Out of 10,157 struts evaluated within 1,117 cross-sections from 30 remaining patients, 302 were classified as malapposed (2.9 %). Only in eight patients the rate of malapposed struts was >5 %. Protruding residual thrombus formations after PCI were apparent significantly more often in patients referred for invasive treatment due to ACS compared with non-ACS patients (ACS vs. non-ACS patients: 47.5 % cross-sections vs. 37.2 % cross-sections, *p* < 0.001). Three-dimensional reconstruction of OCT pullbacks documented 45 SB of diameter >1.5 mm covered by the scaffold struts, out of which 41 were available for evaluation. The different patterns of compartmentalization were recorded as follows: 20 % non-jailed SB (*n* = 8), 17 % V-type jailed SB (*n* = 7), 39 % T-type jailed SB (*n* = 16), 17 % H-type jailed SB (*n* = 7), and 7 % double T-type jailed SB (*n* = 3).

### Follow-up

Complete follow-up was available in 92 % patients (*n* = 98). The mean follow-up period was 147 ± 119 days. The rate of patient-related outcome (MACE) was 6.1 % (*n* = 6) and device-related outcome 2.0 % (*n* = 2) (Table 3).

**Table 3:** Outcomes

Total no. of patients	Outcomes (*n* = 98)
Device-related end point	2 (2.0)
Cardiac death	1 (1.0)
Target vessel Ml	2 (2.0)
TLR	2 (2.0)
Patient-related end point	6 (6.1)
All-cause death	2 (2.0)
Any reinfarction	3 (3.1)
Any revascularization	4 (4.1)

Depicted are counts, *n*, incidence (%)

Two patients died, one of cardiovascular cause. One patient underwent urgent CABG, while two patients had definite scaffold thrombosis (one acute and one subacute  scaffold thrombosis). The acute scaffold thrombosis has been documented by OCT [20]. No possible/probable scaffold thrombosis was documented in this subset.

## Discussion

Our preliminary experience with the use of second-generation everolimus-eluting BVS in a wide patient spectrum suggests that these novel scaffolds are feasible and efficacious even in unstable patients and complex lesions. Indeed, the unrestricted use of BVS at both institutions included also elderly patients, those with ACS and/or complex lesions including chronic total occlusions, massive calcifications, restenosis, and bifurcation lesions.

Currently, the most commonly used second-generation DES with advanced design features shows a substantial reduction in adverse outcomes when compared with first-generation paclitaxel- or sirolimus-eluting stents and in particular BMS. The permanent metal structure of current stents prevents the recoil process and negative remodeling. However, the metal alloy remains a source of inflammation predisposing to neoatherosclerosis and ST and prevents normal coronary vasomotion during episodes of increased demand [21, 22].

These remaining disadvantages of metal scaffolding prompted the exploration of better treatment strategies in patients with CAD. The potential advantage of bioabsorbable scaffolds is the fact that the foreign body only transiently remains present, allowing for proper healing and later normal coronary vasomotion. Furthermore, implantation of bypass grafts remains possible, if later on required. Indeed, the current lactic acid BVS resolves within 2−3 years [23]. Also, first experiences including a few patients with simple lesions were published using other scaffolds, i.e., absorbable metal scaffolds [24, 25]. In contrast to earlier bioabsorbable magnesium stents [24], the everolimus-eluting BVS retains important features of second-generation DES. The bioabsorption of the lactic acid scaffold may result in a reduction of chronic inflammation processes, thereby reducing vessel irritation and restoration of natural vascular function and positively influencing the blood velocity as well as endothelial shear stress [26].

Second-generation BVS has been designed to overcome limitations of metal stents including permanent double layers of struts, struts at the side branch ostium, and stent under deployment due to vasoconstriction or thrombus sequestration. However, data regarding the potential use of BVS in bifurcation lesions and in emergency cases are still scarce. No original data are available, i.e., to affirm the performance of BVS to pass the struts of a previously implanted scaffold in the main branch. In our work we present the feasibility of the BVS T-stenting technique regardless of its relatively high crossing profile.

Furthermore, BVS may be an option in patients with stent restenosis requiring stent-in-stent implantation, contraindications to prolonged DAPT, and those with long lesions [27]. Although, the scaffold seems to be covered by endothelial cells early on after implantation, the requirement of prolonged DAPT after BVS implantation is still uncertain. In patients with in-stent restenosis, no-flow limiting dissections, and acceptable non-stent like primary result, the use of drug-coated balloons (DCB) is one of the possible therapy options. DCB could be also a considered in case of contraindications for prolonged DAPT. Unfortunately, POBA by itself has many limitations including, i.e., flow-limiting dissections. Moreover, DCB could not control the elastic recoil after PCI when no stent was implanted. Thus, BVS might be more efficient in thrombotic lesions, after plaque ruptures, in flow-limiting dissections, and especially in young patients.

Although currently not recommended, BVS may represent the treatment of choice also in patients with CTO, in which commonly the entire vessel is affected by the atherosclerotic process. However, data regarding the safety and performance in a wide spectrum of population is still limited. Particularly, scaffold overlap has been hypothesized to be prone to the delayed healing process, due to a thickness of minimum 300 µm. In our representative case of CTO treated with BVS, full strut coverage was documented after 5 months of follow-up (Fig. 3) despite the strut malapposition rate of 7.1 % within three scaffolds of a summarized length of 74 mm.

Of note, in our real-world population, the MACE rate was comparable with that reported for the unrestricted use of second-generation DES [28]. The high rate of ST (2.1 %) observed in our population may be related to a suboptimal lesion preparation and/or malapposed scaffolds [20]. In patients with ST, the scaffold was indeed incorrectly implanted as documented by OCT. In our experience, ST occurred in an elderly patient referred for primary PCI due to ACS, where BVS was implanted in a heavily calcified LAD [20].

Currently, the applicability of BVS in calcified lesions is a matter of debate. Most physicians are still skeptical with regard to the device deliverability and radial strength [29]. Indeed, unlike the currently used DES, the deliverability of BVS seems more challenging due to the strut thickness of 150 μm and crossing profile of 1.4 mm [30]. However, the new BVS (ABSORB 1.1) currently on the market has several modifications as compared to the previous one (ABSORB 1.0) investigated in cohort A. Also, the polymer underwent several modifications related to the polymer processing, improved device retention, and scaffold design to give more prolonged radial support [31]. Stents should be designed to withstand the difference between the transluminal and intraluminal pressures up to 175 mmHg. Thus, concerning the safety issues, the minimal acceptable collapse pressure for stents is 300 mmHg [32, 33]. Furthermore, the lumen of the vessel appears to stabilize approximately 3 months after PCI [34].

Another downside of current BVS is the fact that they are radiolucent, except for platinum markers. Thus, in cases with long and/or calcified lesions, accurate lesion preparation including the use of noncompliant balloons appears advisable. Furthermore, subsequent intravascular imaging should be obtained to avoid incomplete scaffold apposition. Potential malapposition can be immediately sealed by accurate balloon post dilatation. Post dilatation, however, should be performed with caution, since overexpansion may fracture struts and disrupt BVS [35]. Despite the wide patient spectrum in this study, the result with regard to strut malapposition was comparable with the OCT substudy of cohort B, where no complex patients were enrolled (*p* = 0.13) [36].

The main limitation of the study was its observational nature, based on the double-center cohort. We did not include a control group and the follow-up period differed between patients. The follow-up was not prespecified and no minimal observation time period was required. DES were additionally implanted in 26 patients, which could have impact on outcome. The implantation of DES was left to the operator`s discretion.

## Conclusions

In summary, our study demonstrates the feasibility and good performance of BVS in an unselected population including patients with ACS and complex coronary anatomy. The use of BVS may be feasible in patients at high risk of restenosis, particularly after extensive stent implantation of target vessel and with a history of diabetes mellitus. Nonetheless, in high-risk patients, i.e., referred for primary PCI due to STEMI and thrombotic lesion including left main and ostial LAD, we suggest an adequate lesion preparation and the use of intravascular imaging to prevent malapposition and device-related adverse events.

## Electronic supplementary material

Below is the link to the electronic supplementary material.

Supplementary material 1 (AVI 3868 kb)

Supplementary material 2 (AVI 2272 kb)

Supplementary material 3 (AVI 3823 kb)

Supplementary material 4 (AVI 4594 kb)

Supplementary material 5 (AVI 2584 kb)

Supplementary material 6 (AVI 4831 kb)

Study flow chart (TIFF 2932 kb)
