# Supplementary figures and images for: Feasibility of second-generation bioresorbable vascular scaffold implantation in complex anatomical and clinical scenarios
Source: Clin Res Cardiol. 2014 Aug 31;104(2):124–35. doi: 10.1007/s00392-014-0757-4 (PMC4315475; doi:10.1007/s00392-014-0757-4)

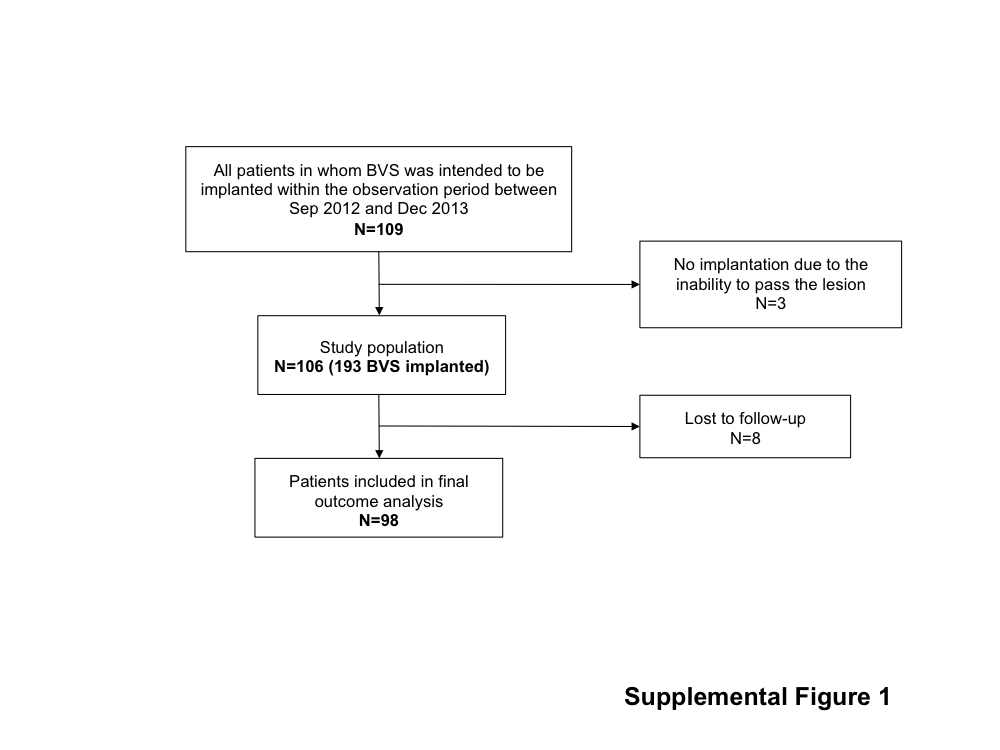

Supplement: Supplementary file 7 — Study flow chart (TIFF 2932 kb) [file 392_2014_757_MOESM7_ESM.tif]
